# Dysregulation of *miR*-*200s* clusters as potential prognostic biomarkers in acute myeloid leukemia

**DOI:** 10.1186/s12967-018-1494-7

**Published:** 2018-05-21

**Authors:** Jing-dong Zhou, Liu-chao Zhang, Ting-juan Zhang, Yu Gu, De-hong Wu, Wei Zhang, Ji-chun Ma, Xiang-mei Wen, Hong Guo, Jiang Lin, Jun Qian

**Affiliations:** 1 Department of Hematology, Affiliated People’s Hospital of Jiangsu University, 8 Dianli Rd., Zhenjiang, 212002 Jiangsu People’s Republic of China; 2 The Key Lab of Precision Diagnosis and Treatment of Zhenjiang City, Zhenjiang, Jiangsu People’s Republic of China; 3 Jingjiang College of Jiangsu University, Zhenjiang, Jiangsu People’s Republic of China; 4 Department of Hematology, The Third People’s Hospital of Kunshan City, Kunshan, Jiangsu People’s Republic of China; 5 Laboratory Center, Affiliated People’s Hospital of Jiangsu University, 8 Dianli Rd., Zhenjiang, 212002 Jiangsu People’s Republic of China

**Keywords:** *miR*-*200*, Expression, Prognosis, Acute myeloid leukemia

## Abstract

**Background:**

Increasing studies showed that *miR*-*200* family (*miR*-*200s*) clusters are aberrantly expressed in multiple human cancers, and *miR*-*200s* clusters function as tumor suppressor genes by affecting cell proliferation, self-renewal, differentiation, division and apoptosis. Herein, we aimed to investigate the expression and clinical implication of *miR*-*200s* clusters in acute myeloid leukemia (AML).

**Methods:**

RT-qPCR was performed to detect expression of *miR*-*200s* clusters in 19 healthy donors, 98 newly diagnosed AML patients, and 35 AML patients achieved complete remission (CR).

**Results:**

Expression of *miR*-*200a/200b/429* cluster but not *miR*-*200c/141* cluster was decreased in newly diagnosed AML patients as compared to healthy donors and AML patients achieved CR. Although no significant differences were observed between *miR*-*200s* clusters and most of the features, low expression of *miR*-*200s* clusters seems to be associated with higher white blood cells especially for *miR*-*200a*/*200b*. Of the five members of *miR*-*200s* clusters, low expression of *miR*-*200b/429/200c* was found to be associated with lower CR rate. Logistic regression analysis further revealed that low expression of *miR*-*429* acted as an independent risk factor for CR in AML. Based on Kaplan–Meier analysis, low expression of *miR*-*200b/429/200c* was associated with shorter OS, whereas *miR*-*200a/141* had a trend. Moreover, multivariate analysis of Cox regression models confirmed the independently prognostic value of *miR*-*200b* expression for OS in AML.

**Conclusions:**

Expression of *miR*-*200a/200b/429* cluster was frequently down-regulated in AML, and low expression of *miR*-*429* as an independent risk factor for CR, whereas low expression of *miR*-*200b* as an independent prognostic biomarker for OS.

**Electronic supplementary material:**

The online version of this article (10.1186/s12967-018-1494-7) contains supplementary material, which is available to authorized users.

## Background

Acute myeloid leukemia (AML) is a highly heterogeneous malignant hematological disorder with complex molecular pathophysiology. Although the treatment strategies against AML have been updated in the past decades, the majority of patients eventually succumb to relapse after induction chemotherapy [1]. Clinical outcome of AML remains unsatisfactory especially in those with specific karyotypes/biomarkers such as inv(3)(q21q26.2), t(6;9)(p23; q34), 11q abnormalities other than t(9;11), -5/del(5q), -7, *TP53* mutations, *FLT3*-ITD mutations, *C*-*KIT* mutations, *WT1* overexpression, and *BAALC* overexpression [2–4]. The development of effective therapeutic options against AML relies on mechanistic understanding of AML biology, especially in molecular regulators of AML pathogenesis and molecular predictor of AML prognosis [5].

MicroRNAs, a class of small (19–22 nucleotides) single-stranded RNAs, negatively regulate various genes by targeting 3′-untranslated region (3′-UTR) of mRNAs, thereby facilitating translational silencing or degradation of targeted genes [6]. Mounting evidences have implicated that microRNAs play crucial roles in regulating many fundamental and biological processes including cancer development [7]. Moreover, microRNAs have been reported as novel biomarkers for diagnosis and prognosis, and regarded as potential therapeutic targets in AML [8]. For instance, recent studies implicated that several microRNAs such as *miR*-*216b*, *miR*-*362*-*5p*, *miR*-*217*, and *miR*-*193b* were prognosis-related predictors in AML and may involve in AML biology [9–12].

The *miR*-*200* family (*miR*-*200s*) clusters includes five members (*miR*-*200a*, *miR*-*200b*, *miR*-*200c*, *miR*-*141*, and *miR*-*429*) and can be divided into two clusters (*miR*-*200a/b/429* cluster and *miR*-*200c/141* cluster) based on chromosomal location (chromosome 1p36 and chromosome 12p13) [13]. Numerous studies showed that *miR*-*200s* clusters are aberrantly expressed in multiple human cancers, and *miR*-*200s* clusters function as tumor suppressor genes by affecting cell proliferation, self-renewal, differentiation, division and apoptosis [14]. Although the tumor-suppressive roles of *miR*-*200s* clusters have also been reported in solid tumors with prognostic value [14, 15], the expression and clinical implication of *miR*-*200s* clusters in AML remains poorly revealed.

In this study, we investigated expression of *miR*-*200s* clusters in AML patients except for acute promyelocytic leukemia (APL), and found that low expression of *miR*-*200s* clusters acted as potential prognostic biomarkers in AML.

## Methods

### Patients and treatment

A total of 98 de novo AML patients except for APL and 19 healthy donors were enrolled in this study. Bone marrow (BM) was collected from all the patients at diagnosis time as well as 35 patients at complete remission (CR) time. AML was diagnosed based on the French–American–British (FAB) and 2016 revised World Health Organization (WHO) criteria [16, 17]. All the patients received chemotherapy as reported [18]. Induction chemotherapy therapy was 1–2 courses of daunorubicin combined with cytarabine. Subsequent consolidation treatment after CR for younger patients included high-dose cytarabine, mitoxantrone with cytarabine, and homoharringtonine combined with cytarabine, whereas for older patients received in an individualized manner decided by the physicians, such as CHG protocol (cytarabine, homoharringtonine, and G-CSF). This study was approved by the Ethics Committee of the Affiliated People’s Hospital of Jiangsu University, and written informed consents were informed and signed by all participants in accordance with the Declaration of Helsinki Principles.

### Cytogenetic analysis and mutation detection

BM cells were harvested after 1–3 days of unstimulated culture in RPMI 1640 medium (BOSTER, Wuhan, China) containing 20% fetal calf serum (ExCell Bio, Shanghai, China). Cytogenetics for AML patients were analyzed at the newly diagnosis time by conventional R-banding method and karyotype risk was classified according to reported previously [19, 20]. Hotspot mutations in *NPM1*, *C*-*KIT*, *DNMT3A*, *N/K*-*RAS*, *IDH1/2*, *U2AF1*, *SRSF2* and *SETBP1* were detected by high-resolution melting analysis [21–25], whereas mutations in *FLT3*-ITD and *CEBPA* were examined by DNA sequencing [26].

### RNA isolation and reverse transcription

BM mononuclear cells (BMMNCs) were extracted as reported using Lymphocyte Separation Medium (Absin, Shanghai, China) [27]. According to the manufacturer’s protocols, RNA was extracted from BMMNCs using the mirVana miRNA isolation kit (Ambion, Austin, TX, USA), and was synthesized to cDNA by reverse transcription using MiScript Reverse Transcription Kit (Qiagen, Duesseldorf, Germany).

### Real-time quantitative PCR

The level of *miR*-*200s* clusters was detected by real-time quantitative PCR (RT-qPCR) using miScript SYBR green PCR kit (Qiagen, Duesseldorf, Germany). The primers were *miR*-*200s* specific (Additional file 1: Table S1) and the manufacturer-provided miScript universal primer (Qiagen, Duesseldorf, Germany). The programs for RT-qPCR reactions were performed as reported [28]. *U6* small nuclear RNA was selected as the endogenous normalizer detected by RT-qPCR using 2× SYBR Green PCR Mix (Multisciences, Hangzhou, China). Relative *miR*-*200s* level was calculated by 2^−ΔΔCT^ method. The healthy donors that possessed the minimal ΔCT between *miR*-*200s* (each member) and *U6* expression was selected as control, and was defined as 100% expression.

### Statistical analysis

Mann–Whitney’s U test was carried to compare the difference of continuous variables between two groups, whereas Pearson Chi square analysis/Fisher exact test were applied to compare the difference of categorical variables between two groups. The impact of *miR*-*200s* clusters expression on overall survival (OS) was analyzed by Kaplan–Meier analysis, and Cox regression models (univariate and multivariate analyses) were further used to determine the independently prognostic value of *miR*-*200s* cluster expression. The effect of *miR*-*200s* clusters expression on CR was determined by Logistic regression analysis (univariate and multivariate analyses). All tests were two sided, and *P *< 0.05 was defined as statistically significant. SPSS software 20.0 and GraphPad Prism 5.0 was used to conduct the statistical analyses in this study.

## Results

### Expression of miR-200s in AML

**Fig. 1:**
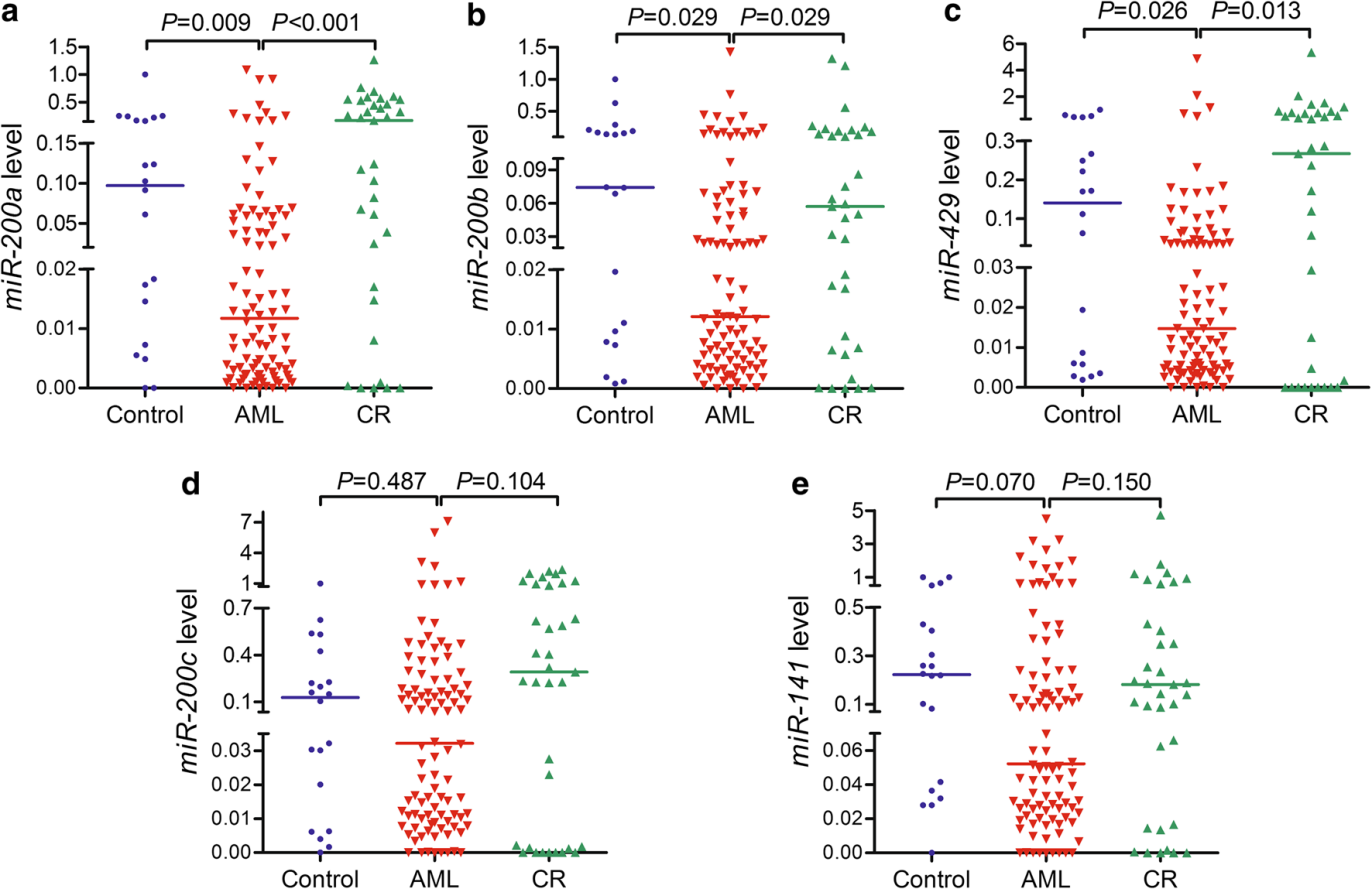
Expression of *miR*-*200s* clusters in controls, newly diagnosed AML patients and AML patients achieved CR. **a** For *miR*-*200a*; **b** For *miR*-*200b*; **c** For *miR*-*429*; **d** For *miR*-*200c*; **e** For *miR*-*141*. The distributions of the *miR*-*200s* clusters expression in controls, newly diagnosed AML patients and AML patients achieved CR were presented with scatter plots. The median level of *miR*-*200s* clusters expression in each group was shown with horizontal line

We analyzed *miR*-*200s* clusters expression in BM from 19 healthy donors, 98 AML patients, and 35 AML patients achieved CR by RT-qPCR. As presented in Fig. 1, expression of *miR*-*200a/200b/429* clusters but not *miR*-*200c/141* clusters was significantly decreased in AML patients as compared to healthy donors and AML patients achieved CR.

### Relationship between miR-200s and clinical features in AML

To investigate clinical implication of *miR*-*200s* clusters expression, the whole-cohort patients were classified into two groups (high and low *miR*-*200s* clusters expression) based on the median level of each member of *miR*-*200s* clusters, respectively. We analyzed the association between each member of *miR*-*200s* clusters expression and clinic-pathologic features including gender, age, white blood cell (WBC) counts, hemoglobin content, platelet counts, blasts (%), FAB subtypes, karyotypes, and common gene mutations. As shown in Table 1, no significant differences were observed between *miR*-*200s* clusters expression and most of the features. However, low expression of *miR*-*200s* clusters seems to be associated with higher WBC counts especially for *miR*-*200a*/*200b* (*P *= 0.001 and 0.041, respectively). In addition, low expression of *miR*-*200a* was related to male, whereas low expression of *miR*-*141* was correlated with higher hemoglobin content (*P *= 0.013 and 0.024, respectively).

**Table 1:** Correlation of *miR*-*200s* cluster expression with clinical/laboratory features in AML patients

Patient’s features	*miR*-*200a* expression			*miR*-*200b* expression			*miR*-*429* expression			*miR*-*200c* expression			*miR*-*141* expression		
	Low (n = 49)	High (n = 49)	*P*	Low (n = 49)	High (n = 49)	*P*	Low (n = 49)	High (n = 49)	*P*	Low (n = 49)	High (n = 49)	*P*	Low (n = 49)	High (n = 49)	*P*
Sex (male/female)	36/13	23/26	0.013	32/17	27/22	0.409	31/18	28/21	0.680	32/17	27/22	0.409	31/18	28/21	0.680
Age (years)	58 (21–81)	61 (18–87)	0.842	60 (18–81)	59 (18–87)	0.831	60 (21–81)	59 (18–87)	0.741	60 (18–81)	59 (18–87)	0.743	60 (18–81)	59 (18–87)	1.000
WBC (× 10^9^/L)	38.7 (1.3–528.0)	9.6 (1.1–130.2)	0.001	35.5 (1.3–528.0)	9.6 (1.1–130.2)	0.041	34.5 (1.3–528.0)	13.3 (1.1–130.2)	0.099	34.9 (1.3–528.0)	13.2 (1.1–130.2)	0.094	35.5 (1.3–528.0)	10.2 (1.1–116.6)	0.090
Hemoglobin (g/L)	78 (53–138)	76.5 (32–144)	0.085	80 (53–138)	76.5 (32–144)	0.330	77 (32–138)	78 (34–134)	0.940	84 (53–138)	76 (32–144)	0.124	88 (53–138)	74.5 (32–144)	0.024
Platelets (× 10^9^/L)	37 (3–447)	47 (4–264)	0.558	37 (3–447)	46 (4–264)	0.460	36 (3–447)	47 (4–264)	0.512	30 (5–447)	49 (3–264)	0.233	40 (5–125)	46 (3–447)	0.769
BM blasts (%)	60% (20–99%)	58% (20–95%)	0.892	63% (20–99%)	58% (21–95%)	0.651	60% (20–98%)	59% (20–99%)	0.757	63% (20–99%)	54% (20–95%)	0.226	62% (20–98%)	57% (20–99%)	0.598
FAB subtypes			0.660			0.945			0.681			0.827			0.900
M0	1	0		1	0		0	1		1	0		1	0	
M1	4	2		3	3		3	3		2	4		2	4	
M2	26	24		23	27		22	28		23	27		24	26	
M4	12	15		14	13		15	12		15	12		15	12	
M5	6	6		7	5		8	4		7	5		6	6	
M6	0	2		1	1		1	1		1	1		1	1	
Karyotypes			0.982			0.813			0.606			0.948			0.578
Normal	25	28		23	30		23	30		24	29		24	29	
t(8;21)	5	5		5	5		6	4		5	5		6	4	
+ 8	2	1		2	1		2	1		2	1		3	0	
− 5/5q-	1	2		2	1		2	1		2	1		2	1	
− 7/7q-	1	0		1	0		0	1		1	0		1	0	
t(9;22)	1	0		1	0		0	1		1	0		1	0	
Complex	8	7		9	6		10	5		8	7		7	8	
Others	6	5		6	5		6	5		6	5		5	6	
No data	0	1		0	1		0	1		0	1		0	1	
Gene mutations															
*CEBPA* (±)	7/33	6/38	0.765	5/38	8/33	0.376	8/36	5/35	0.555	7/34	6/37	0.768	6/37	7/34	0.768
*NPM1* (±)	5/35	4/40	0.730	3/40	6/35	0.307	4/40	5/35	0.730	5/36	4/39	0.735	4/39	5/36	0.735
*FLT3*-ITD (±)	6/34	3/41	0.298	6/37	3/38	0.484	6/38	3/37	0.488	5/36	4/39	0.735	6/37	3/38	0.484
*C*-*KIT* (±)	1/39	1/43	1.000	1/42	1/40	1.000	1/43	1/39	1.000	1/40	1/42	1.000	1/42	1/40	1.000
*N/K*-*RAS* (±)	4/36	6/38	0.741	3/40	7/34	0.190	5/39	5/35	1.000	6/35	4/39	0.515	6/37	4/37	0.739
*IDH1/2* (±)	1/39	3/41	0.618	3/40	1/40	0.616	4/40	0/40	0.118	4/37	0/43	0.052	2/41	2/39	1.000
*DNMT3A* (±)	4/36	2/42	0.418	4/39	2/39	0.676	4/40	2/38	0.678	3/38	3/40	1.000	3/40	3/38	1.000
*U2AF1* (±)	2/38	2/42	1.000	1/42	3/38	0.354	1/43	3/37	0.343	2/39	2/41	1.000	3/40	1/40	0.616
*SRSF2* (±)	1/39	3/41	0.618	1/42	3/38	0.354	1/43	3/37	0.343	1/40	3/40	0.616	1/42	3/38	0.354
*SETBP1* (±)	2/38	0/44	0.224	2/41	0/41	0.494	2/42	0/40	0.495	2/39	0/43	0.235	2/41	0/41	0.494
CR (±)	16/33	23/26	0.215	14/35	25/24	0.038	14/35	25/24	0.038	14/35	25/24	0.038	15/34	24/25	0.098

*WBC* white blood cells, *BM* bone marrow, *FAB* French–American–British classification, *CR* complete remission

### Prognostic value of miR-200s in AML

To observe the impact of *miR*-*200s* clusters expression on clinical outcome in AML, we first determined the association of each member of *miR*-*200s* clusters expression with CR. Of the five members of *miR*-*200s* clusters, low expression of *miR*-*200b/429/200c* was found to be associated with lower CR rate (Table 1, all *P *= 0.038). Additionally, Logistic regression analysis was further performed to confirm and verify the effect of *miR*-*200s* clusters’ expression on CR, and revealed low expression of *miR*-*429* as an independent risk factor for CR in AML (Table 2, *P *= 0.023).

**Table 2:** Univariate and multivariate analyses of variables for overall survival in AML patients

Variables	Complete remission				Overall survival			
	Univariate analysis		Multivariate analysis		Univariate analysis		Multivariate analysis	
	OR (95% CI)	*P*	OR (95% CI)	*P*	HR (95% CI)	*P*	HR (95% CI)	*P*
*miR*-*200a*	0.548 (0.242–1.244)	0.150	1.029 (0.285–3.722)	0.965	0.662 (0.415–1.022)	0.082	1.425 (0.651–3.120)	0.376
*miR*-*200b*	0.384 (0.167–0.885)	0.025	0.823 (0.199–3.401)	0.788	0.511 (0.319–0.819)	0.005	0.524 (0.305–0.902)	0.020
*miR*-*429*	0.384 (0.167–0.885)	0.025	0.331 (0.128–0.858)	0.023	0.558 (0.350–0.891)	0.015	0.820 (0.325–2.073)	0.675
*miR*-*200c*	0.384 (0.167–0.885)	0.025	0.977 (0.149–6.400)	0.981	0.606 (0.380–0.965)	0.035	0.649 (0.190–2.217)	0.491
*miR*-*141*	0.460 (0.201–1.050)	0.065	0.594 (0.192–1.833)	0.364	0.695 (0.437–1.104)	0.123	1.152 (0.582–2.279)	0.684
Age	4.229 (1.742–10.266)	0.001	4.555 (1.715–12.095)	0.002	2.046 (1.282–3.266)	0.003	1.732 (1.033–2.902)	0.037
WBC	2.367 (1.015–5.520)	0.046	1.846 (0.715–4.767)	0.206	2.002 (1.253–3.199)	0.004	1.560 (0.925–2.629)	0.095
Karyotype	3.108 (1.338–7.220)	0.008	2.862 (1.164–7.042)	0.022	1.875 (1.295–2.715)	0.001	1.874 (1.210–2.902)	0.005
*CEBPA* mutations	0.526 (0.160–1.731)	0.290			0.870 (0.413–1.829)	0.713		
*NPM1* mutations	0.833 (0.207–3.358)	0.798			1.200 (0.516–2.793)	0.672		
*FLT3*-ITD mutations	0.833 (0.207–3.358)	0.798			0.935 (0.403–2.170)	0.876		
*C*-*KIT* mutations	0.673 (0.041–11.150)	0.783			0.479 (0.066–3.458)	0.465		
*N/K*-*RAS* mutations	3.048 (0.605–15.343)	0.177			1.311 (0.621–2.770)	0.478		
*IDH1/2* mutations	Undetermined	0.999			4.671 (1.637–13.326)	0.004	6.662 (1.757–25.268)	0.005
*DNMT3A* mutations	1.391 (0.240–8.057)	0.712			1.590 (0.634–3.987)	0.323		
*U2AF1* mutations	Undetermined	0.999			2.791 (0.987–7.890)	0.053	5.130 (1.714–15.355)	0.003
*SRSF2* mutations	Undetermined	0.999			1.934 (0.693–5.400)	0.208		
*SETBP1* mutations	0.673 (0.041–11.150)	0.783			0.637 (0.088–4.613)	0.656		

*OR* odd ratio, *HR* hazard ratio, *CI* confidence interval. Variables including *miR*-*200s* cluster expression (Low vs. High), age (≤ 60 vs. > 60 years), WBC (≥ 30 × 10^9^ vs. < 30 × 10^9^/L), karyotype (favorable vs. intermediate vs. poor), and gene mutations (mutant vs. wild-type). Multivariate analysis includes variables with *P *< 0.200 in univariate analysis

**Fig. 2:**
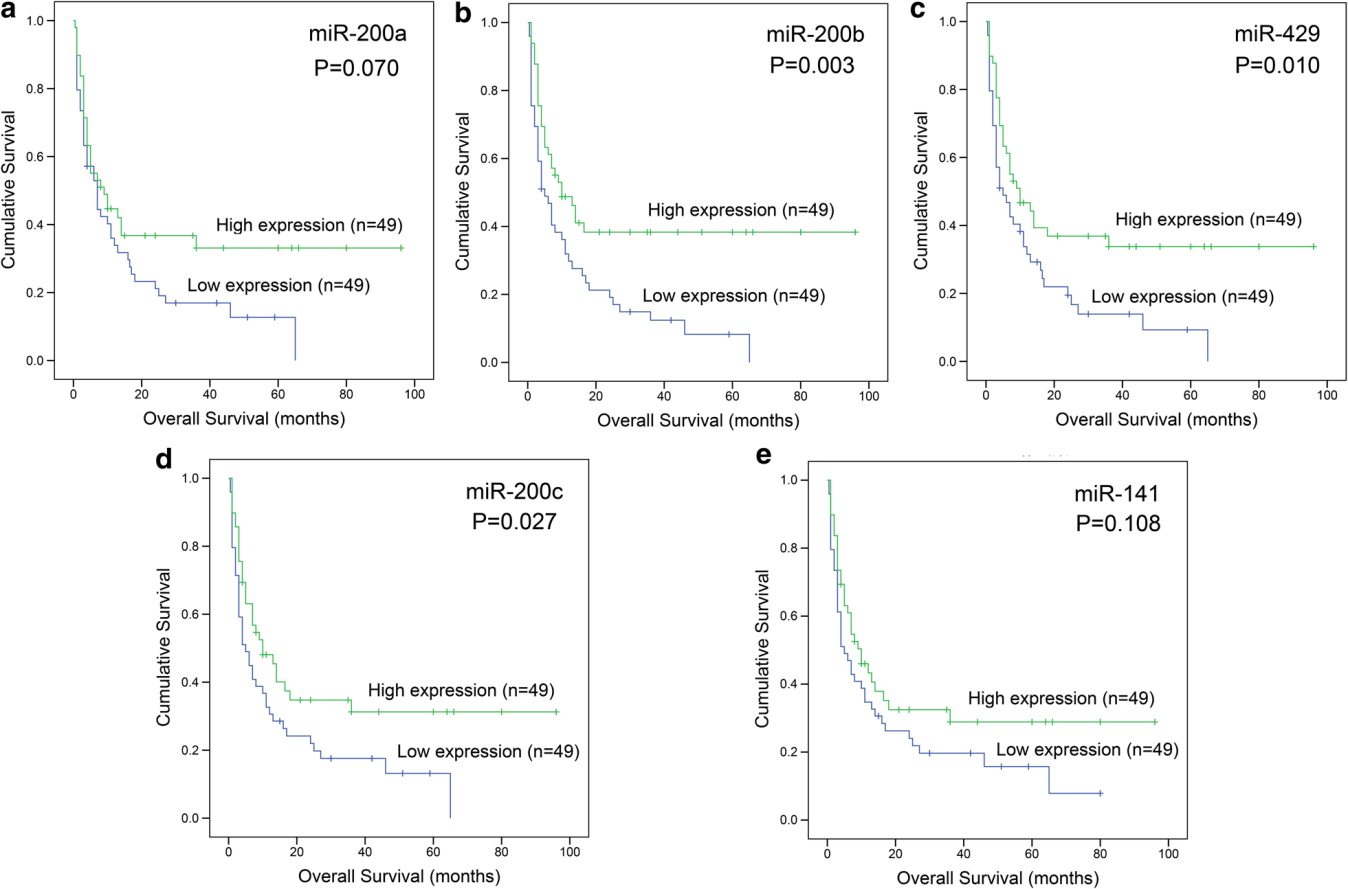
Prognostic value of each member of *miR*-*200s* clusters expression in AML. **a** For *miR*-*200a*. **b** For *miR*-*200b*. **c** For *miR*-*429*. **d** For *miR*-*200c*. **e** For *miR*-*141*. Overall survival analyzed between two groups based on median level of each member of *miR*-*200s* clusters, and performed by Kaplan–Meier methods

**Fig. 3:**
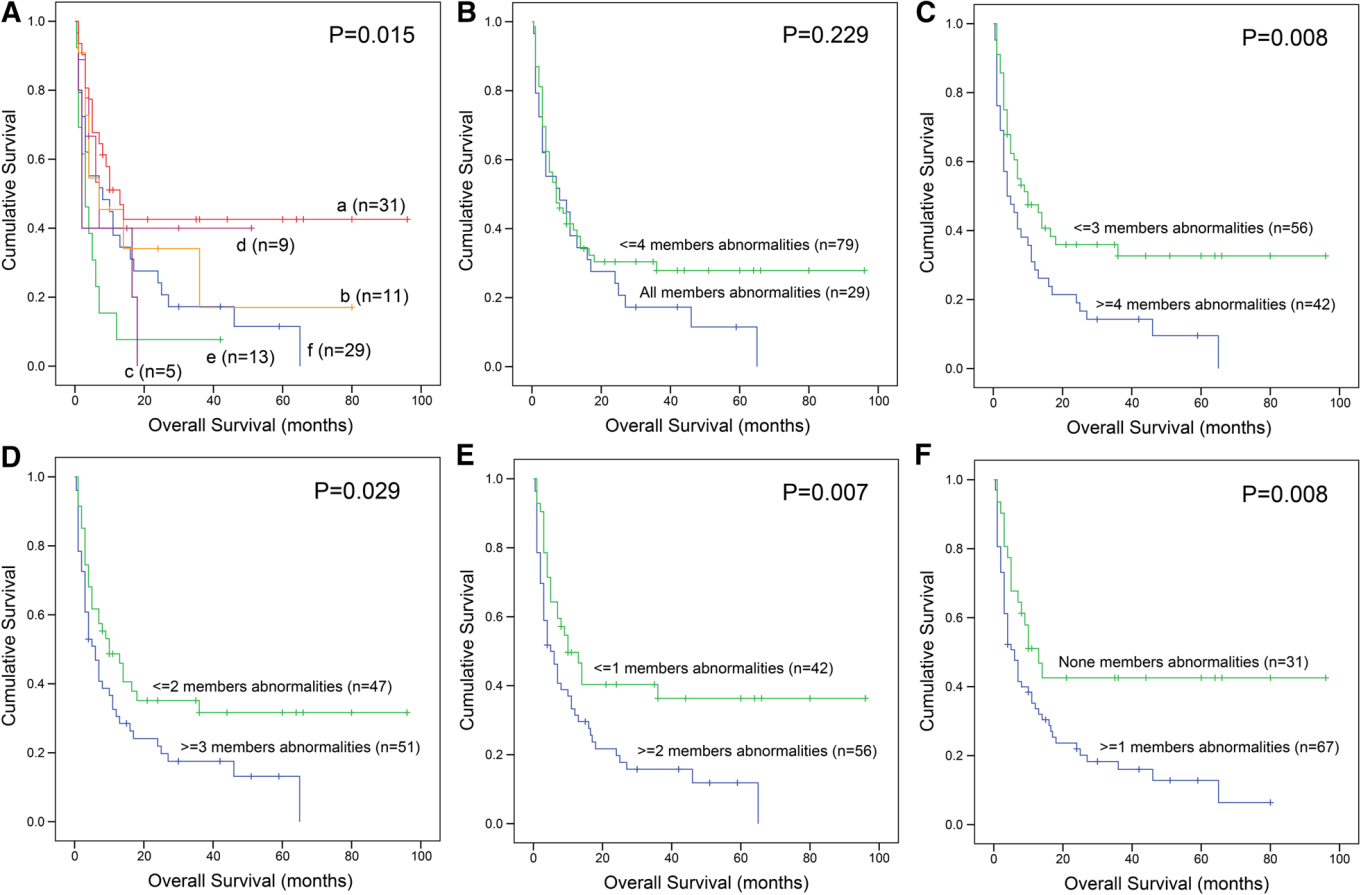
Prognostic value of composite members of *miR*-*200s* clusters expression in AML. **A** Overall survival (OS) analyzed among AML patients with different numbers of abnormal *miR*-*200s* clusters expression; a: without *miR*-*200s* clusters abnormalities; b: with only one member of *miR*-*200s* clusters abnormalities; c: with two members of *miR*-*200s* clusters abnormalities; d: with three members of *miR*-*200s* clusters abnormalities; e: with four members of *miR*-*200s* clusters abnormalities; f: with all members of *miR*-*200s* clusters abnormalities. **B** OS analyzed between two groups (all members of *miR*-*200s* clusters abnormalities vs. equal or less than four members of *miR*-*200s* clusters abnormalities). **C** OS analyzed between two groups (equal or more than four members of *miR*-*200s* clusters abnormalities vs. equal or less than three members of *miR*-*200s* clusters abnormalities). **D** OS analyzed between two groups (equal or more than three members of *miR*-*200s* clusters abnormalities vs. equal or less than two members of *miR*-*200s* clusters abnormalities). **E** OS analyzed between two groups (equal or more than two members of *miR*-*200s* clusters abnormalities vs. equal or less than one member of *miR*-*200s* clusters abnormalities). **F** OS analyzed between two groups (equal or more than one member of *miR*-*200s* clusters abnormalities vs. without any members of *miR*-*200s* clusters abnormalities)

We next evaluated the correlation of each member of *miR*-*200s* clusters expression with survival. Based on Kaplan–Meier analysis, low expression of *miR*-*200b/429/200c* was associated with shorter OS, whereas *miR*-*200a/141* had a trend (Fig. 2). In addition, we also analyzed the impact of composite members of *miR*-*200s* clusters expression on OS by Kaplan–Meier analysis as shown in Fig. 3.

Since *miR*-*200s* clusters expression was associated with well-established prognostic factor such as WBC counts, we further conducted a Cox regression model adjusting for prognosis-related factors (age, WBC counts, karyotypic classifications, and gene mutations) for OS. Results showed that low expression of *miR*-*200b* acted as an independent prognostic biomarker for OS (*P *= 0.020, Table 2).

## Discussion

In the current study, we for the first time investigated expression of *miR*-*200s* clusters in AML, and revealed that most of the members of *miR*-*200s* clusters were down-regulated in de novo AML patients. Recently, Li et al. revealed that introduction of a pre-*miR*-*200c* reduced the expression of ZEB2 protein and inhibited the proliferation of human leukemia cell lines (HL-60, MOLM-13, and THP-1), and mouse *miR*-*200c* significantly impaired the proliferation of mouse leukemia cells [29]. Taken together, these results emphasized the crucial role of *miR*-*200s* clusters in leukemogenesis. Although the biological role of *miR*-*200s* clusters in AML was less studied, tumor suppressor roles of *miR*-*200s* clusters have been identified in a variety of human solid cancers, such as bladder cancer, gastric cancer, colorectal cancer, breast cancer, ovarian cancer, endometrial cancer, pancreatic cancer, gliomas, hepatocellular carcinoma, and lung cancer [14, 30]. The *miR*-*200s* clusters were reported as key inhibitors of epithelial-to-mesenchymal transition by directly targeting transcriptional repressors of E-cadherin, ZEB1, and ZEB2 [13]. Moreover, *miR*-*200s* clusters also played crucial roles in the repression of cancer stem cells self-renewal and differentiation, modulation of cell division and apoptosis, and reversal of chemoresistance [14, 30]. Notably, in some other hematological malignancies, expression or biological role of *miR*-*200s* clusters has been preliminary studied. For instance, Choi et al. reported that *miR*-*200c* was decreased in patients with myelodysplastic syndrome (MDS) [31]. González-Gugel et al. revealed that down-regulation of mmu-*miR*-*30a* and mmu-*miR*-*141* as well as hsa-*miR*-*193b* clearly contributed to enhance the expression of *Smoothened* (*SMO*) gene in mouse and human lymphomas and, subsequently, to activate the GLI/Hh signalling [32].

In addition to basic research before, it has been noted that low expression of *miR*-*200s* clusters could correlate with adverse clinical outcome and serve as a prognostic biomarker for various cancer patients [15]. Although the potential prognostic value of *miR*-*200s* clusters in several human cancers remains controversial, a recent meta-analysis demonstrated that lower tissue expression of *miR*-*200s* clusters’ members were associated with poor OS and progression-free survival, whereas lower expression of circulating *miR*-*200s* clusters’ members were correlated with favorable prognosis [15]. From our study, we showed the negative effect of low expression of *miR*-*200s* clusters on AML chemotherapy response and survival. Moreover, multivariate analysis showed that low expression of *miR*-*429* as an independent risk factor for CR, whereas low expression of *miR*-*200b* as an independent prognostic biomarker for OS in AML. Due to some limitations in this study (such as patients numbers, treatment regimens, and single center), prospective studies are needed to verify our results before *miR*-*200s* clusters expression could be used routinely as a promising biomarker for risk stratification in AML.

## Conclusion

Expression of *miR*-*200a/200b/429* cluster was frequently down-regulated in AML, and low expression of *miR*-*429* as an independent risk factor for CR, whereas low expression of *miR*-*200b* as an independent prognostic biomarker for OS.

## Additional file

**Additional file 1: Table S1.** The primer sequences for *miR*-*200s* clusters.

## Abbreviations

AML: acute myeloid leukemia
3′-UTR: 3′-untranslated region
APL: acute promyelocytic leukemia
BM: bone marrow
CR: complete remission
BMMNCs: BM mononuclear cells
FAB: French–American–British
WHO: World Health Organization
RT-qPCR: real-time quantitative PCR
OS: overall survival
WBC: white blood cell
