# Supplementary material for: Dysregulation of miR-200s clusters as potential prognostic biomarkers in acute myeloid leukemia
Source: J Transl Med. 2018 May 21;16:135. doi: 10.1186/s12967-018-1494-7 (PMC5963159; doi:10.1186/s12967-018-1494-7)
Supplement: Supplementary file 1 — Additional file 1: Table S1. The primer sequences for miR-200s clusters. [file 12967_2018_1494_MOESM1_ESM.docx]

**Additional file 1: Table S1. The primer sequences for *miR-200s* clusters.**

| Premier name | Premier sequences |
| --- | --- |
| *miR-200a* specific | TAACACTGTCTGGTAACGATGT |
| *miR-200b* specific | TAATACTGCCTGGTAATGATGA |
| *miR-429* specific | TAATACTGTCTGGTAAAACCGT |
| *miR-200c* specific | TAATACTGCCGGGTAATGATGGA |
| *miR-141* specific | TAACACTGTCTGGTAAAGATGG |
